# Metacognition in wild Japanese macaques: cost and stakes influencing information-seeking behavior

**DOI:** 10.1007/s10071-024-01851-z

**Published:** 2024-03-05

**Authors:** Lorraine Subias, Noriko Katsu, Kazunori Yamada

**Affiliations:** Graduate School of Human Sciences, Osaka University, 1-2 Yamadaoka, Suita, Osaka Japan

**Keywords:** Metacognition, Information seeking, Japanese macaques, Tube task

## Abstract

Metacognition allows us to evaluate memories and knowledge, thus enabling us to distinguish between what we know and what we do not. Studies have shown that species other than humans may possess similar abilities. However, the number of species tested was limited. Testing ten free-ranging Japanese macaques (*Macaca fuscata*) on a task in which they had to find food hidden inside one of the four opaque tubes, we investigated whether these subjects would seek information when needed. The monkeys could look inside the tubes before selecting one. We varied three parameters: the baiting process, the cost that monkeys had to pay to look inside the tubes, and the reward at stake. We assessed whether and how these parameters would affect the monkeys’ tendency to look inside the tube before selecting one. When they were not shown which tube contained the reward, nine monkeys looked significantly more frequently in at least one condition. Half of them tended to reduce their looks when the cost was high, but only when they already knew the location of the reward. When a high-quality reward was at stake, four monkeys tended to look more inside the tubes, even though they already knew the reward’s location. Our results are consistent with those of rhesus macaques, suggesting that metacognitive-like abilities may be shared by Cercopithecidae, and that, at least some monkeys may be aware of their lack of knowledge.

**Supplementary Information:**

The online version contains supplementary material available at 10.1007/s10071-024-01851-z.

## Introduction

Metacognition allows us to assess what we know and recognize when we do not know enough, which, in turn, allows us to optimize information-seeking before deciding, balancing the need to gather more information, the cost of acquiring it, and the risk of not doing so (Beran and Smith 2011). Non-human animals also encounter situations in which they do not have sufficient information, such as not knowing where to find food or who they might come across. Having the ability to recognize a lack of knowledge or an ambiguous situation may allow animals to adopt more efficient strategies to search for food, avoid danger, or find solutions to problems more effectively.

Various protocols have been developed to investigate metacognition in nonhuman primates, some testing subjects ability to escape difficult trials (Brown et al. 2017; Hampton 2001; Smith et al. 1995; Templer and Hampton 2012), others, to bet on the certainty of their choices (Beran et al. 2015; Kornell et al. 2007; Morgan et al. 2014) or even to seek additional information when needed (Templer 2019). Based on an information-seeking paradigm, the tube task proposed by Call and Carpenter (2001) requires little training and is often considered to represent a more naturalistic situation than other types of protocols. A reward is hidden in one of several tubes (or under a cup in some versions) such that the subject cannot see which one contains the reward without bending down and peering into the tube opening (or under the cups). In the seen/visible trials, the experimenter inserted the reward inside the tube while the subject was watching it. In the unseen/hidden trials, the baiting took place behind a barrier so the subject could not know which tube held the reward. The subject was allowed to look inside the tubes and/or select one tube. The selection of an empty tube resulted in no reward. Human children and great apes have shown similar tendencies to look inside the tubes before selection in “unseen” trials (Call 2010; Call and Carpenter 2001). By contrast, in “seen” trials, they readily selected a tube without looking.

Various explanations that do not require metacognition have been proposed for this information-seeking behavior. In experiments involving escape response paradigms, it has been noted that monkeys might rely on external cues to monitor their behavior without truly being aware of their knowledge state (Crystal and Foote 2009; Jozefowiez et al. 2009; Smith et al. 2009). Although the tube task requires much less training, it is still possible that subjects learn, during testing, to associate observable cues with outcomes, such as looking inside the tubes. For instance, the opaque barrier used in “unseen” trials could serve as a cue, indicating to the subject that they should look, since looking in this condition would have been more rewarded in previous trials than not looking.

Some researchers have also argued that animals simply display generalized search behavior when they do not have a representation of the reward location (Carruthers 2008; Hampton et al. 2004; Hampton 2009; Kornell et al. 2007). According to this generalized search hypothesis, subjects explore their environment until a reward is detected.

Another plausible explanation is response competition (Crystal and Foote 2011; Hampton 2009). Competing drives, such as drives to search or reach for food (or “go where something good is,” Crystal and Foote 2011) might guide an animal’s behavior. When food is spotted (as in a visible baiting trial), the drive to reach for it, and, to reach for the tube in which the food is known to be present, would presumably be strong. By contrast, when food is not spotted, the drive to reach for a particular tube or container is weaker. Hence, the drive to search for food randomly developed through past experience and trial and error, is more likely to occur (Carruthers 2008; Hampton et al. 2004; Hampton 2009).

According to Call (2010), these three hypotheses are weakened by two factors in the case of the great apes. First, subjects sometimes selected the correct tube after having looked inside the empty tube only (on 17–34% of the trials depending on the species, Call and Carpenter 2001; Call 2005). In a similar study using three cups, Marsh and MacDonald (2012a) also observed that 2/3 orangutans reduced unnecessary looking when the location of the reward could be inferred. Additionally, Beran et al. (2013) conducted an experiment refuting the notion that apes simply engage in search behavior when they lack information about the location of food. Second, in the tube task, although subjects looked inside the tubes more often in the hidden trials than in the visible trials, they looked inside the tube in the visible trials from time to time. Call compared this behavior to a person checking multiple times for their passport before a trip, as the cost of doing so is low compared to the cost of forgetting their passport (Call named this the “passport effect”). To test this effect, several studies presented great apes with modified versions of the tube task in which the cost of seeking information was manipulated as well as the value of the reward at stake.

Great apes were more likely to look inside the tubes before choosing when the cost of looking was lower and high stakes were involved, a pattern that challenged previous non-metacognitive explanations (Call 2010; Gazes et al. 2023; Marsh and MacDonald 2012b; Mulcahy 2016). If apes’ looks are driven by response competition mechanisms, an increased cost should not have a differential effect on looking behavior, and the use of a high-value reward should create a stronger drive to reach the tube in visible trials rather than to look. This result does not fit well with the assumption that animals adopt a generalized food search strategy neither. Call concluded that ape-looking response seems to be a function of at least three factors: the cost of looking inside the tube, the value of the reward, and the state of the information—a combination that creates an information processing system that possesses complexity, flexibility, and control: three of the features of metacognition, as argued by Smith (2009). Although several studies have tested monkey species in the information-seeking paradigm using the tube task, none have yet attempted to manipulate the cost of seeking and stakes to determine whether monkeys would show the same complexity and flexibility in their seeking behavior as great apes.

In addition, investigations of memory awareness have been limited to very few species. In Cercopithecidae, only baboons (*Papio papio*; Malassis et al. 2015), lion-tailed macaques (*Macaca silenus*; Marsh 2014), and rhesus macaques (*Macaca mulatta*; Basile et al. 2015; Beran and Smith 2011; Brady and Hampton 2021; Hampton et al. 2004) have been tested in an information-seeking paradigm with positive results. In Platyrrhini, investigations have mainly been restricted to capuchin monkeys (*Cebus apella*) and have found contradictory results (Basile et al. 2008, 2015; Beran and Smith 2011; Fujita 2009; Hampton et al. 2004; Malassis et al. 2015; Marsh 2014; Paukner et al. 2006; Vining and Marsh 2015). Lemurs have also been tested, with results providing little evidence of metacognition (Taylor et al. 2020). It remains unclear whether the failure of capuchins and lemurs indicates that metacognition evolved selectively or more strongly after Platyrrhini and Cercopithecidae lineages diverged, or if the methodologies employed to test those species were not suitable (Smith et al. 2018). To gain a clearer view of the evolutionary emergence of metacognitive capacities, addressing the question of species differences is a necessary step toward mapping the phylogenetic distribution of these abilities. Testing more species may help us determine whether the capacity to monitor one’s own behavior based on memory content is a general one shared by most animals, or if it evolved in response to specific ecological or social selection pressures.

To further investigate this topic, we tested free-ranging Japanese macaques (*Macaca fuscata*) using the tube task. Three members of this species have previously participated in a study examining the effects of prefrontal cortex lesions on their performance in a spatial discrimination task with an escape response (Kwok et al. 2019). While it is reasonable to assume that Japanese macaques and rhesus macaques will show similar metacognitive performance because of their close phylogenetic relationships (Hamada and Yamamoto 2010), there is currently no research clearly demonstrating this.

Another presupposition that needs to be supported by the data is the assumption that laboratory populations are representative of their wild relatives. The study of animal populations maintained in the laboratory is a common practice in biological sciences to draw conclusions about the ecology, cognitive abilities, and evolution of wild animals. However, the performances displayed by animals in the laboratory may be quite different from what they can do in the wild. Among many other effects, laboratory conditions may lead to captive animals being more motivated or habituated to solving cognitive tasks than wild animals (Pritchard et al. 2016). For example, captive kea (*Nestor notabilis*) learned how to lift a tube more readily than wild kea (Gajdon et al. 2004). Wild-spotted hyenas (*Crocuta crocuta*) were also less successful at approaching and solving novel tasks than captive hyenas (Benson-Amram et al. 2013). It cannot be excluded that the metacognitive performances observed in captive animals are the result of overtrained animals that are used to learning quickly and solving all types of cognitive tasks. To the best of our knowledge, only one study has attempted to assess metacognition, specifically information-seeking, in wild macaques living in their natural habitats (Rosati and Santos 2016). This study used a one-shot foraging task, where each monkey completed a single trial. Like their captive counterparts, free-ranging rhesus monkeys tended to seek information when they were ignorant. Further research is needed to confirm these findings, as well as to examine the potential influence of other factors to control for alternative non-metacognitive explanations, using a protocol that includes repeated testing of the same subjects.

Japanese macaques have been habituated to human presence since 1952 through the creation of various feeding sites in Japan and are well suited for experimental studies on cognition in the wild (Nakagawa et al. 2010). The ability to easily observe and identify individual macaques at these sites, as well as the opportunity for long-term studies and interpopulation comparisons, makes them ideal candidates for such research.

The first goal of this study was to examine whether wild Japanese macaques would tend to seek information when they are ignorant, as has been reported for captive rhesus and lion-tailed macaques tested in an information-seeking paradigm. We presented the macaques with the tube task and confronted them with two types of trials: where they knew which tube contained a reward (obvious trials) and where they did not (ambiguous trials). The second goal of this study was to provide further insight into the mechanisms that might guide monkeys’ information-seeking behaviors. If information-seeking behavior is not based on metacognition, there are two prevalent opposing hypotheses: associative learning and response competition mechanisms. To exclude these possibilities, we manipulated the cost of seeking information and the stakes in the experiment. Call (2010) tested apes using two tubes and he modified tube position (straight or oblique) to manipulate the cost of looking. However, preliminary observations revealed that our monkeys were unlikely to look inside the tubes when using only two tubes. Instead, like rhesus and capuchin monkeys (Beran et al. 2014), they appeared sensitive to the probability of being rewarded by chance. Consequently, we increased the number of tubes (four) and adjusted the apparatus height to manipulate the cost. To manipulate the stakes, we used different types of food rewards following Call’s protocol.

We hypothesized that if Japanese monkeys were able to monitor their behavior based on their knowledge state, they would look inside the tubes more often in ambiguous than in obvious trials. In addition, if their information-seeking behavior relied solely on external cues (associative learning), we could expect no effect of cost and stakes or a similar effect in both obvious and ambiguous conditions. However, if their information-seeking behavior relied on responses competition mechanisms, these monkeys would look even less when a preferred reward was at stake, compared to a less preferred reward if they knew the reward’s location.

## Methods

### Subjects

The tests were conducted on a free-ranging group of Japanese macaques (*Macaca fuscata*) in Awajishima, Hyôgo Prefecture, Japan (34°14′41.5″N, 134°52′59.6″E). This group has been artificially provisioned by the Awajishima Monkey Center since 1967. Wheat and soybeans were provided three times daily. At the time of the study, the group size was estimated at 450. Observational studies have been conducted on this population since 1978 (Nakamichi et al. 1983), and experimental studies have investigated cooperative behavior (Kaigaishi et al. 2019).

This study was conducted from February to July 2022, from 9:30 a.m. to 5 p.m. daily, in the feeding area, where all the subjects ranged freely. They were free to participate by approaching and manipulating the apparatus. Although we started to train approximately 30 monkeys, only ten came and stayed at the apparatus long enough to complete the experiment. The participants included nine males and one female, all adults (> 7 years old; Table 1). With the exception of a few, their exact age and rank were unknown.

**Table 1:** Subjects included in the study

Name	Age (years)	Sex	Rank	Location 1	Location 2
Gaara	15 ~ 19	M	un	0	225
Gattsu	17	M	6	425	0
Izuna	~ 10	M	un	6	233
Kikuhime	~ 20	M	7	251	0
Manta	15	M	15	17	200
Paku	15 ~ 19	M	12	40	240
Puriko09	13	F	2	92	154
Spot	15 ~ 20	M	un	335	0
Tim	15 ~ 20	M	un	239	0
Yubisashi	~ 20	M	un	114	27

~ means an approximate age, “un” stands for unknown and numbers under location 1 and 2 columns indicates the number of trials that were run at each location

### Testing location

Two testing locations were established. One was in a wire-meshed hut, where visitors could feed monkeys sweet potatoes and peanuts. Many monkeys gathered around the hut, and only high-ranking individuals were able to approach. To allow lower-ranking monkeys to participate, a second testing location was set up in another hut away from visitors and with fewer monkeys.

During testing at both locations, the experimenter stood inside the hut with the apparatus, while the subject monkey stood outside the hut and interacted with the apparatus through a wire mesh. At location 1, the subject sat on a wooden platform located 103 cm above the ground, whereas at location 2, it sat directly on the ground.

Four subjects were exclusively tested at location 1, one at location 2, and the remaining five were tested at both locations depending on the opportunity (Table 1).

### Apparatus

**Fig. 1:**
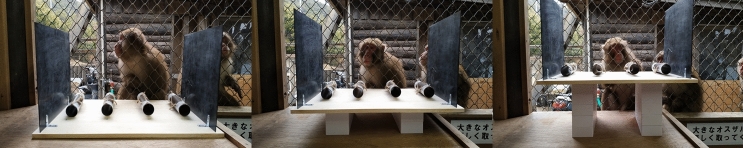
From left to right: apparatus at levels 1, 3, and 6 (levels 2, 4, and 5 are not represented)

The monkeys were confronted with the tube task developed by Call and Carpenter (2001), using a four-tube apparatus similar to that used by Hampton et al. (2004). The apparatus consisted of a wooden board (600 mm × 400 mm), four PVC tubes, and a transparent PVC screen (550 mm × 320 mm). Tubes, 45 mm in diameter and 200 mm in length, were placed approximately 55 mm apart, parallel to each other, and fixed on a wooden board from one end (on the subject’s side) to be lifted and tipped over. The screen was placed in front of the tubes, perpendicular to the board. The final 60 mm of the screen was covered with an opaque black band that occluded the hole in the tube. Therefore, when the screen was lowered, the subjects could see the tubes from above, but not inside the tubes. A piece of string was attached to the tubes for the monkeys to tip over by pulling on the string. White PVC boxes (55 × 85 × 43 mm) that could be piled up were placed under the wooden board to raise the apparatus to different levels, from level 1 (no boxes used; the apparatus was directly placed on the table) to level 6 (215 mm high, Fig. 1). The apparatus was elevated by 43 mm at each level. Two identical apparatuses were built for use at locations 1 and 2.

### Procedure

#### Pre-training

The procedure was initiated whenever a monkey arrived in front of the apparatus and interacted with the tubes without interference from others. Pre-training consisted of habituating the monkeys to the apparatus and teaching them to grab the tubes or pull on the strings to make a tube tip over and obtain the food they may contain. During this phase, the monkeys were able to look inside the tubes at any time. Once they had learned to choose a baited tube by tipping it, we proceeded with the training.

#### Training

Prior to the test, the monkeys were trained to watch the experimenter (E) randomly place a food reward in one of the four tubes and pick one after a short delay. If the monkey selected an empty tube, E showed the monkey where the reward was before removing it and repeating the trial. During this phase, veil plugs were inserted into the tubes to prevent the monkeys from looking, while allowing the reward to pass through. The idea was to train the monkeys to pay attention to baiting and rely on their memory to find a reward. The apparatus was placed at levels 1 and 6. The subjects received blocks of 12 trials, with no limit per day, first at Level 1. When they reached an 80% success rate at Level 1 on two consecutive blocks, they were trained at Level 6 until they reached an 80% success rate on two consecutive blocks.

#### Test

**Fig. 2:**
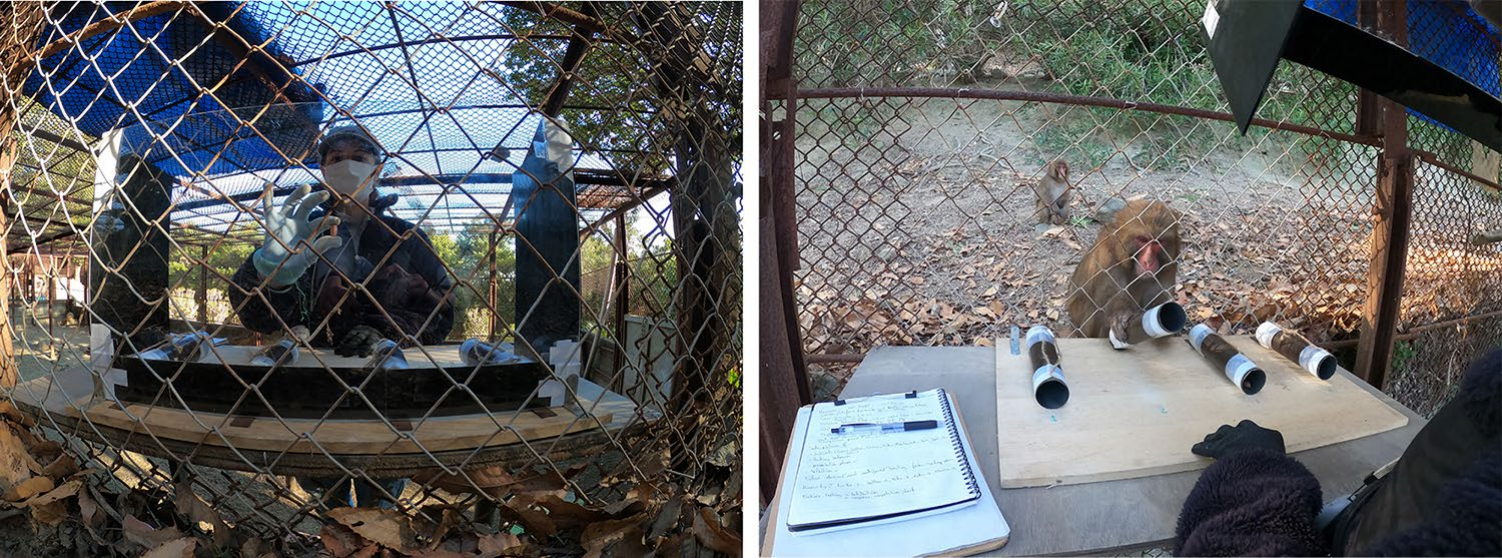
Presentation phase from monkey’s side (left picture) and choice phase (right picture) from experimenter’s side at location 2

The basic procedure was the same as that used for the training. E presented a food reward to the subject and placed it inside one of the tubes. During the presentation phase, the transparent screen was lowered in front of the tubes to prevent the participant from touching or looking inside the tubes (Fig. 2). The food location was randomized with the constraint that it was never placed in the same tube for more than two consecutive trials.

After placing the reward, E immediately removed the screen for the participant to look inside the tubes and/or choose one by tipping it over (Fig. 2). If the subject chose the tube containing the reward, they received it. If they chose an empty tube, E took the reward away, showing it to the subject before starting a new trial.

We varied three parameters: (1) the baiting process, (2) the quality of the food reward at stake, and (3) the cost the subject had to pay to look inside the tubes.

The baiting could either be:

*Obvious*: After presenting the reward, E inserted her finger into the baited tube only, making it obvious which tube contained the reward.

*Ambiguous*: After presenting the reward, E manipulated it by passing it from one hand to the other so that the subject would no longer know which hand contained the reward. E then inserted her fingers into every tube while discretely leaving the reward in one tube. The pattern was always the same: First, E inserted her fingers simultaneously into the two outer tubes and then into the two middle tubes.

Traditionally, in the tube task, an opaque panel is used to block subjects' view of the baiting and create “unknown” trials. However, because we were planning to test the same monkeys in a second experiment requiring an opaque panel, we employed an “ambiguous” baiting process here to prevent the monkeys from associating the panel with a looking response.

The food quality (and so, the stakes) could either be:

*Low stakes*: Monkeys less preferred food was used as a reward: a piece of sweet potato or carrot, depending on the individual.

*High stakes*: Monkeys most preferred food was used as a reward: a peanut.

See Online Resource 1 for detail about how monkeys food preferences were assessed.

To manipulate the cost, we adjusted the apparatus height; the higher the apparatus, the easier it was for the monkey to look inside the tubes. Two possible conditions were tested:

*Low cost*: The apparatus was placed at a level at which the subject would be willing to look inside the tubes often (between 50 and 75% of the trials).

*High cost*: The apparatus was placed at a level at which the subject did not look too often (between 25 and 50% of the trials).

Each subject was tested 12 times under eight conditions (Table 2). The conditions were tested by alternating them in a random manner. When possible (i.e., when the monkey stayed long enough), subjects received two trials of each condition/day, with a minimum of eight trials/day and a maximum of 16 trials/day (excluding titration trials; see Titration procedure). In addition, we tested two control conditions.

**Table 2:** Conditions tested

	Baiting	Look cost	Stakes
Condition 1a	Obvious	Low	Low
Condition 1b	Ambiguous	Low	Low
Condition 2a	Obvious	High	Low
Condition 2b	Ambiguous	High	Low
Condition 3a	Obvious	Low	High
Condition 3b	Ambiguous	Low	High
Condition 4a	Obvious	High	High
Condition 4b	Ambiguous	High	High

*Control1*: Under this condition, baiting was obvious, food quality was low, and the veil plugs were on, preventing the monkey from looking inside the tubes even during the choice phase. This condition served to assess the monkeys’ working memory performance.

*Control2*: In this condition, baiting was ambiguous, food quality was low, and the veil plugs were on, preventing the monkey from looking inside the tubes. This condition served to check whether the monkeys truly did not know the location of the reward in the ambiguous condition.

The subjects received four control trials (two trials of condition control 1 + two trials of control 2) at the end of each test session when possible (i.e., when the monkey stayed until the end of the session) until we reached a minimum of 12 trials per control condition.

#### Titration procedure

Since the motivation to look inside the tubes differed across subjects, and even across days for the same subject, we had to use a titration procedure at each test session to determine the level of use for low- and high-cost conditions. The titration procedure prevented ceiling and floor effects from masking the differential-looking behavior in obvious and ambiguous trials.

The procedure was as follows: We started by giving four trials (half obvious, half ambiguous) to the subject with the apparatus at the last level used in the previous session or level 6 if it was the first session. If (a) the monkey looked inside the tubes in every trial, the apparatus was lowered from one level, and E gave four trials again; (b) the monkey never looked, the apparatus was raised from one level, and E gave four trials; and (c) the monkey made between zero and four looks, the apparatus was kept at the same level, and E gave four more trials. This was repeated until we obtained eight trials each at levels that could qualify for the low-cost condition (50% < looks < 75%) and high-cost condition (25% < looks < 50%).

If the apparatus reached level 1 and the monkey was still looking at more than 50% of the trials, level 1 was chosen as the high-cost level and level 2 as the low-cost level, independent of the monkey’s number of looks. However, if the apparatus reached level 6 and the monkey was still looking at less than 50% of the trials, level 6 was chosen as the low-cost level and level 5 as the high-cost level.

To avoid the order effect from biasing the data, we counterbalanced the conditions we first tested (low- or high-cost conditions). For example, if in the previous session an individual was first tested at level 4 for the low-cost condition and then at level 2 for the high-cost condition, in the next session, the experimenter will start with the apparatus at level 2 and try to complete the testing for the high-cost condition before looking for an appropriate level for the low-cost condition.

Trials used for titration were not considered for the data analysis, as they only reflected ceiling and floor effects.

#### Special training

Among our ten subjects who completed the training and reached the testing, one individual (Paku) never looked inside the tubes for 5.6 sessions of titration attempts. To elicit a look, we made him go through special training. This consisted of 16 ambiguous trials/day sessions at Level 6 until the subject started to look. It underwent two sessions of special training before he started looking inside the tube.

#### Behavior scoring

In each trial, the number and location of looks made in the tubes were noted, as well as whether the correct tube was selected. The experimenter watched and scored the monkeys’ behaviors in real time. Touching a tube or string without lifting the tube was not considered a selection, except for one subject—Gattsu. Being born with severely malformed hands, Gattsu was unable to grab objects. To make this choice, he had to touch the tube with a stump. For others, slightly lifting the tube was scored as the choice.

A look was scored when the subjects lowered their heads and bodies such that their eyes were aligned with the tube opening. As there were four tubes to look into, we noted the monkeys’ looking patterns and identified four strategies. An *efficient* strategy was one in which the subject ended his search after having gathered enough information to find the reward (i.e., after spotting the reward inside a tube or looking inside three empty tubes). In the *insufficient* strategy, the subject ended his search before he had sufficient information to find the reward (i.e., after looking inside only one or two empty tubes). An *excessive* strategy was one in which the subject continued looking even after having all the information he needed to find the reward (i.e., looked into the fourth tube after having looked inside three empty tubes or kept looking after having looked inside the baited tube).

Sessions were recorded on videotape.

#### Data analysis

We analyzed the percentage of trials that examined the responses as a function of the condition. Since the looking proportion did not always follow a normal distribution, we used two-tailed non-parametric Wilcoxon signed-rank tests for group-level comparisons. For individual-level comparisons, we used chi-square tests or Fisher’s exact tests whenever the expected value was < 5.

With the possibility that monkeys will look more when a high-quality reward is at stake, as has been observed with apes (Call 2010), we compared the cost effect using data from the low-quality reward conditions only (Table 2; Conditions 1 and 2).

As for monkey performance, we used binomial tests with a probability of success equal to 0.25, to assess whether the success of the monkeys significantly exceeded chance.

Using video recordings, 20% of the analyzed trials were scored again by the experimenter and a second observer. The reliability between real-time and video observations, as well as interobserver reliability, was assessed using Cohen’s Kappa. The reliability was almost perfect for both comparisons (real-time vs. Video: kappa = 0.97; Observer1 vs. Observer2: kappa = 0.95).

All the analyses were run on Rstudio 4.1.0.

## Results

### Accuracy in finding the reward

**Fig. 3:**
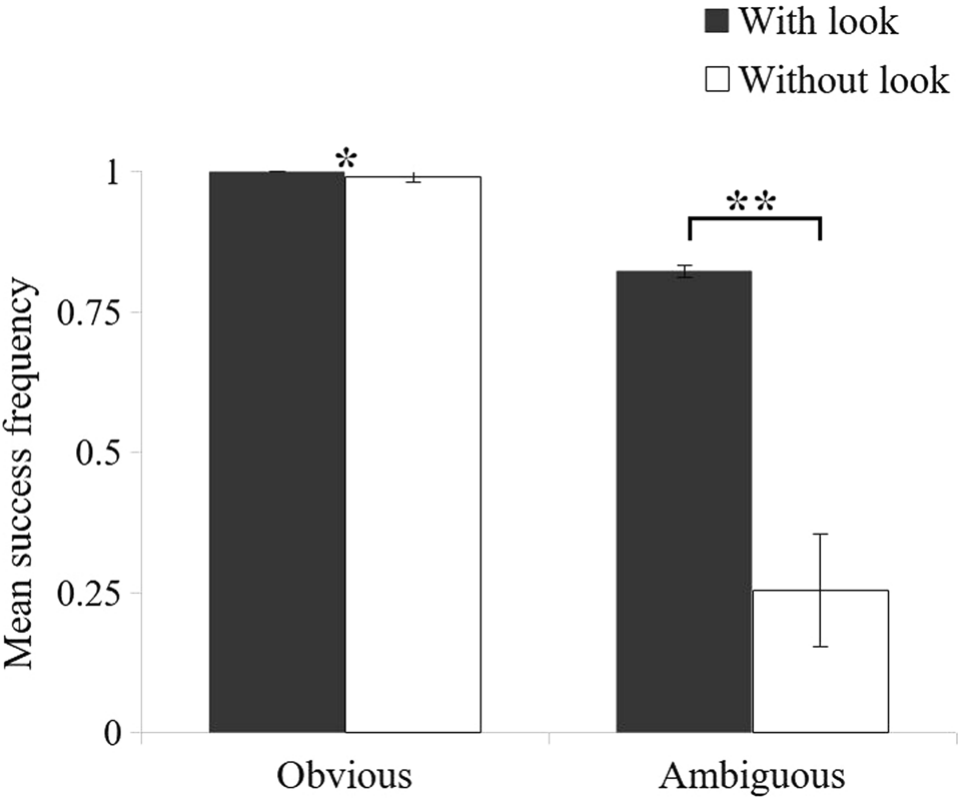
Mean frequency of successful trials as a function of the baiting process. “With look” indicates trials in which monkeys looked inside at least one tube before making their choice, whereas “Without look” indicates trials in which monkeys did not look inside the tubes. Bars represent the standard deviation (SD)

Data from all four test conditions were used to assess monkeys accuracy in finding the reward “With look”. As for monkeys accuracy in finding the reward “Without look”, the data from the test conditions were completed by the data from the control conditions. The monkeys were very good at finding the reward when baiting was obvious, regardless of whether they looked inside the tubes. While there was a statistically significant difference between trials in which they looked and trials in which they did not look (Wilcoxon rank test: *N* = 10, *Z* = − 2.02, *p* = 0.043), there were no meaningful differences in success rate: when looking inside the tubes before choosing, their accuracy was perfect (100 ± 0% of success ± SD) and near perfect without looking (99 ± 1% of success on average ± SD) (Fig. 3). By contrast, when the baiting was ambiguous, monkeys were significantly more accurate when they looked through the tubes before choosing (82 ± 10% of success ± SD) than on trials where they failed to look (25 ± 09% of success ± SD; Wilcoxon rank test: *N* = 10, *Z* = − 2.80, *p* = 0.005).

These results indicate that monkeys needed to look inside the tubes to know the location of the reward in the ambiguous condition, but not in the obvious condition. In addition, to confirm that the monkeys did not know the reward’s location in the ambiguous condition, we checked whether their success frequency exceeded chance. Performance on ambiguous trials without looks did not significantly exceed chance level for any subject except Gattsu (Online Resource 2, binomial test: success = 30/87, *p* = 0.047). Nevertheless, Gattsu performed significantly better on ambiguous trials in which he looked (69% success) than on which he did not look (34% success, chi-squared test: *χ*^2^(1.168) = 21.83, *p* < 0.001). These results confirm that the method used to create the “unknown” trials was successful.

### Baiting effect

**Fig. 4:**
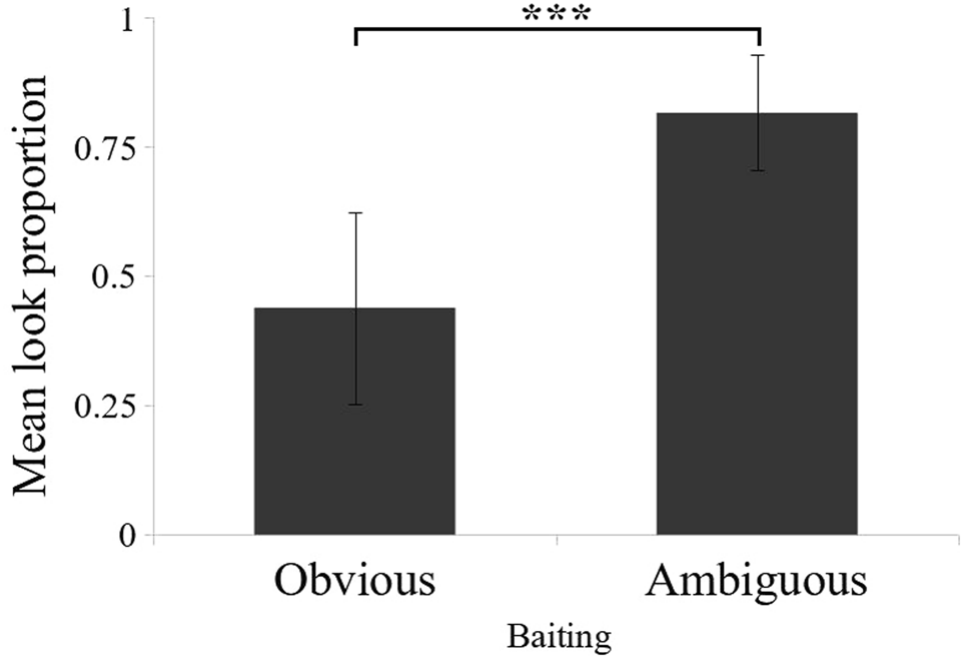
Mean proportion of trials in which subjects made at least one look inside one tube before choosing as a function of the baiting process. Data from conditions 1, 2, 3, and 4 (Table 3) were combined

The subjects looked inside the tubes significantly more often when the baiting was ambiguous (82 ± 11% of trials ± SD) than obvious (44 ± 19% of trials ± SD), when all conditions were combined (Fig. 4, Wilcoxon signed-rank test: *N* = 10, *Z* = − 3.30, *p* < 0.001). Moreover, this pattern was observed at an individual level (Table 3). Eight of our ten monkeys looked significantly more often in the ambiguous condition (Table 4, Obvious vs. Ambiguous). One (Gattsu), looked more often in the ambiguous condition compared to obvious but only when the cost to look was low (Table 3), and this difference reached statistical significance only in condition 3, when a high-quality reward was at stake (Obvious vs. Ambiguous, condition 1: χ^2^(1.74) = 2.96, *p* = 0.09; condition 2: χ^2^(1.65) = 0.028, *p* = 0.87; condition 3: χ^2^(1.66) = 6.28, *p* = 0.012; condition 4: χ^2^(1.71) = 0.40, *p* = 0.53). Only one monkey (Yubisashi) did not seem to be affected by the baiting process and looked in almost every trial, regardless of the condition (89% of trials in obvious, 94% of trials in ambiguous, Fisher test: *p* = 0.49). In the end, nine of our ten monkeys tended to look significantly more often after ambiguous baiting in at least one condition (Table 5). By contrast, none of the monkeys looked significantly more often at obvious trials than at ambiguous trials. These results support the idea that Japanese monkeys adjust their information-seeking behavior based on whether they have seen the reward location.

**Table 3:** The “Success” table shows the number of trials in which subjects succeeded in finding the reward/the total number of trials, in obvious and ambiguous conditions

	Success				Look proportions							
	Obvious		Ambiguous		Condition 1 low cost and stakes		Condition 2 High cost and low stakes		Condition 3 Low cost and high stakes		Condition 4 High cost and stakes	
	With look	Without look	With look	Without look	Obv.	Amb.	Obv.	Amb.	Obv.	Amb.	Obv.	Amb.
GROUP	100% (377/377)	99% (684/690)	82% (572/693)	26% (97/364)	56% (120/220)	92% (198/219)	29% (60/217)	73% (150/215)	54% (114/217)	92% (199/218)	37% (80/218)	69% (142/212)
Gaara	100% (35/35)	98% (58/59)	69% (43/62)	22% (7/32)	57% (12/21)	85% (17/20)	32% (6/19)	81% (17/21)	40% (8/20)	95% (19/20)	41% (9/22)	43% (9/21)
Gattsu	100% (66/66)	99% (101/102)	69% (56/81)	34% (30/87)	57% (21/37)	76% (28/37)	34% (11/32)	36% (12/33)	50% (16/32)	76% (27/34)	49% (18/37)	41% (14/34)
Izuna	100% (27/27)	99% (70/71)	94% (66/70)	28% (7/25)	62% (13/21)	100% (18/18)	20% (4/20)	80% (16/20)	42% (8/19)	95% (18/19)	10% (2/20)	90% (18/20)
Kikuhime	100% (32/32)	99% (66/67)	76% (47/62)	21% (8/39)	60% (12/20)	95% (19/20)	20% (4/20)	82% (18/22)	55% (11/20)	76% (16/21)	25% (5/20)	47% (9/19)
Manta	100% (18/18)	100% (59/59)	79% (46/58)	18% (4/22)	44% (7/16)	88% (15/17)	12% (2/17)	82% (14/17)	40% (6/15)	88% (15/17)	18% (3/17)	82% (14/17)
Paku	100% (18/18)	100% (94/94)	97% (86/89)	09% (2/23)	17% (4/23)	100% (25/25)	14% (3/22)	88% (22/25)	33% (9/27)	96% (24/25)	08% (2/24)	86% (18/21)
Puriko09	100% (47/47)	100% (72/72)	79% (53/67)	25% (12/48)	48% (12/25)	88% (21/24)	24% (6/25)	35% (8/23)	68% (17/25)	96% (22/23)	52% (12/23)	67% (16/24)
Spot	100% (35/35)	100% (73/73)	80% (55/69)	22% (8/36)	52% (12/23)	95% (21/22)	24% (5/21)	74% (14/19)	48% (10/21)	100% (22/22)	42% (8/19)	63% (12/19)
Tim	100% (49/49)	97% (68/70)	86% (72/84)	33% (11/33)	63% (15/24)	92% (22/24)	26% (7/27)	77% (17/22)	63% (15/24)	100% (25/25)	52% (12/23)	83% (20/24)
Yubisashi	100% (50/50)	100% (23/23)	94% (48/51)	42% (8/19)	100% (12/12)	100% (12/12)	86% (12/14)	92% (12/13)	100% (14/14)	92% (11/12)	69% (9/13)	92% (12/13)

“With look” indicates that subjects looked at least once inside one tube before choosing; “without look” indicates that subjects chose a tube without looking. The “Look” table shows the number of trials in which subjects looked inside the tubes before choosing/the total number of trials, for each condition. “Obv.” indicates an obvious baiting, whereas “Amb.” indicates an ambiguous baiting

**Table 4:** Look proportion comparisons results

	Obvious versus ambiguous	Low versus high cost (low stakes)		Low versus high stakes (high cost)		Low versus high stakes (low cost)	
		Obvious	Ambiguous	Obvious	Ambiguous	Obvious	Ambiguous
GROUP (N = 10)	*Z* = − 3.30, *p* < 0.001*	*Z* = − 2.75, *p* = 0.006*	*Z* = − 2.75, *p* = 0.006*	*Z* = − 1.33, *p* = 0.19	*Z* = − 0.35, *p* = 0.73	*Z* = − 0.63, *p* = 0.53	*Z* = − 0.059, *p* = 0.95
Gaara	χ^2^ = 18.40, *p* < 0.001*	χ^2^ = 2.63, *N* = 40 *p* = 0.10	*N* = 41, *p* = 1.00(*f*)	χ^2^ = 0.38, *N* = 41 *p* = 0.54	χ^2^ = 6.46, *N* = 42 *p* = 0.011*	χ^2^ = 1.20, *N* = 41 *p* = 0.27	*N* = 40, *p* = 0.61(f)
Gattsu	χ^2^ = 3.28, *p* = 0.07	χ^2^ = 3.46, *N* = 69 *p* = 0.063	χ^2^ = 11.01, *N* = 70 *p* = 0.0009*	χ^2^ = 1.44, *N* = 69 *p* = 0.23	χ^2^ = 0.16, *N* = 67 *p* = 0.69	χ^2^ = 0.32, *N* = 69 *p* = 0.57	χ^2^ = 0.14, *N* = 71 *p* = 0.71
Izuna	χ^2^ = 54.29, *p* < 0.001*	χ^2^ = 7.41, *N* = 41 *p* = 0.006*	*N* = 38, *p* = 0.11(*f*)	*N* = 40, *p* = 0.66(*f*)	*N* = 40, *p* = 0.66(*f*)	χ^2^ = 1.57, *N* = 40 *p* = 0.21	*N* = 37, *p* = 1.00(f)
Kikuhime	χ^2^ = 21.08, *p* < 0.001*	χ^2^ = 6.67, *N* = 40 *p* = 0.010*	*N* = 42, *p* = 0.35(*f*)	*N* = 40, *p* = 1.00(*f*)	χ^2^ = 5.38, *N* = 41 *p* = 0.020*	χ^2^ = 0.10, *N* = 40 *p* = 0.75	*N* = 41, *p* = 0.18(f)
Manta	χ^2^ = 45.03, *p* < 0.001*	*N* = 33, *p* = 0.057(*f*)	*N* = 34, *p* = 1.00(*f*)	*N* = 34, *p* = 1.00(*f*)	No diff	χ^2^ = 0.045, *N* = 31 *p* = 0.83	No diff
Paku	χ^2^ = 106.42, *p* < 0.001*	*N* = 45, *p* = 1(*f*)	*N* = 50, *p* = 0.23(*f*)	*N* = 46, *p* = 0.66(*f*)	*N* = 46, *p* = 1.00(*f*)	χ^2^ = 1.64, *N* = 50 *p* = 0.20	*N* = 50, *p* = 1.00(f)
Puriko09	χ^2^ = 10.82, *p* = 0.001*	χ^2^ = 3.13, *N* = 50 *p* = 0.077	χ^2^ = 13.81, *N* = 47 *p* < 0.001*	χ^2^ = 1.46, *N* = 48 *p* = 0.23	χ^2^ = 4.78, *N* = 47 *p* = 0.023*	χ^2^ = 2.05, *N* = 50 *p* = 0.15	*N* = 47, *p* = 0.61(f)
Spot	χ^2^ = 32.0, *p* < 0.001*	χ^2^ = 3.73, *N* = 44 *p* = 0.053	*N* = 41, *p* = 0.080(*f*)	χ^2^ = 1.52, *N* = 40 *p* = 0.22	χ^2^ = 0.49, *N* = 38 *p* = 0.49	χ^2^ = 0.091, *N* = 44 *p* = 0.76	*N* = 44, *p* = 1.00(f)
Tim	χ^2^ = 33.24, *p* < 0.001*	χ^2^ = 6.93, *N* = 51 *p* = 0.008*	*N* = 46, *p* = 0.23(*f*)	χ^2^ = 3.63, *N* = 50 *p* = 0.057	*N* = 46, *p* = 0.72(*f*)	No diff	*N* = 49, *p* = 0.23(f)
Yubisashi	*p* = 0.49(*f*)	*N* = 26, *p* = 0.48(*f*)	*N* = 25, *p* = 1.00(*f*)	*N* = 27, *p* = 0.38(*f*)	No diff	*N* = 26, *p* = 1.00(f)	*N* = 24, *p* = 1.00(f)

χ^2^ and *p*-value from Chi-squared tests (individual comparisons, *df* = 1) and Wilcoxon signed rank tests (group comparisons) when comparing look frequencies. Whenever the Chi-squared test provided an expected frequency of < 5, Fisher test was performed instead, as marked (*f*) in the table

**Table 5:** Alternative hypotheses predictions and results summary

	Predictions		Results
	Associative learning	Response competition	
Baiting effect	Look proportion should be: Obv. < Amb.	Look proportion should be: Obv. < Amb.	GROUP/Obv. < Amb.
			9/10 subjects showed a difference between Obv. and Amb.
			1/10 subject showed no difference
Cost effect	Low = High (no effect)		GROUP/Low > High in Obv. and Amb.
	Or		5/10 subjects showed a different effect in Obv. and Amb. (contradict associative learning predictions)
	Low > High in Obv. and Amb. (equal effect)		5/10 subjects showed no effect, or equal effect in Obv. and Amb.
Stakes effect	Low = High (no effect)	Low > High in Obv.	GROUP/Low = High
	Or		3/10 subjects showed an increase between Low and High in Obv. (contradict associative learning and response competition predictions)
	Low < High in Obv. and Amb. (similar increase)		1/10 subject showed a similar increase between Low and High in Obv. and Amb. (contradict response competition prediction)
	Or		6/10 subjects showed no effect (contradict response competition prediction)
	Low > High in Obv. and Amb. (similar decrease)		

Predictions for alternative hypotheses regarding the effects of baiting, cost, and stakes on macaques looking proportions. “Obv.” and “Amb.” respectively refer to the “obvious” and “ambiguous” conditions. The “Results” column indicates main results for the group and provides details on the number of subjects for which the results align or do not align with the predictions made by the alternative hypotheses

### Cost effect

**Fig. 5:**
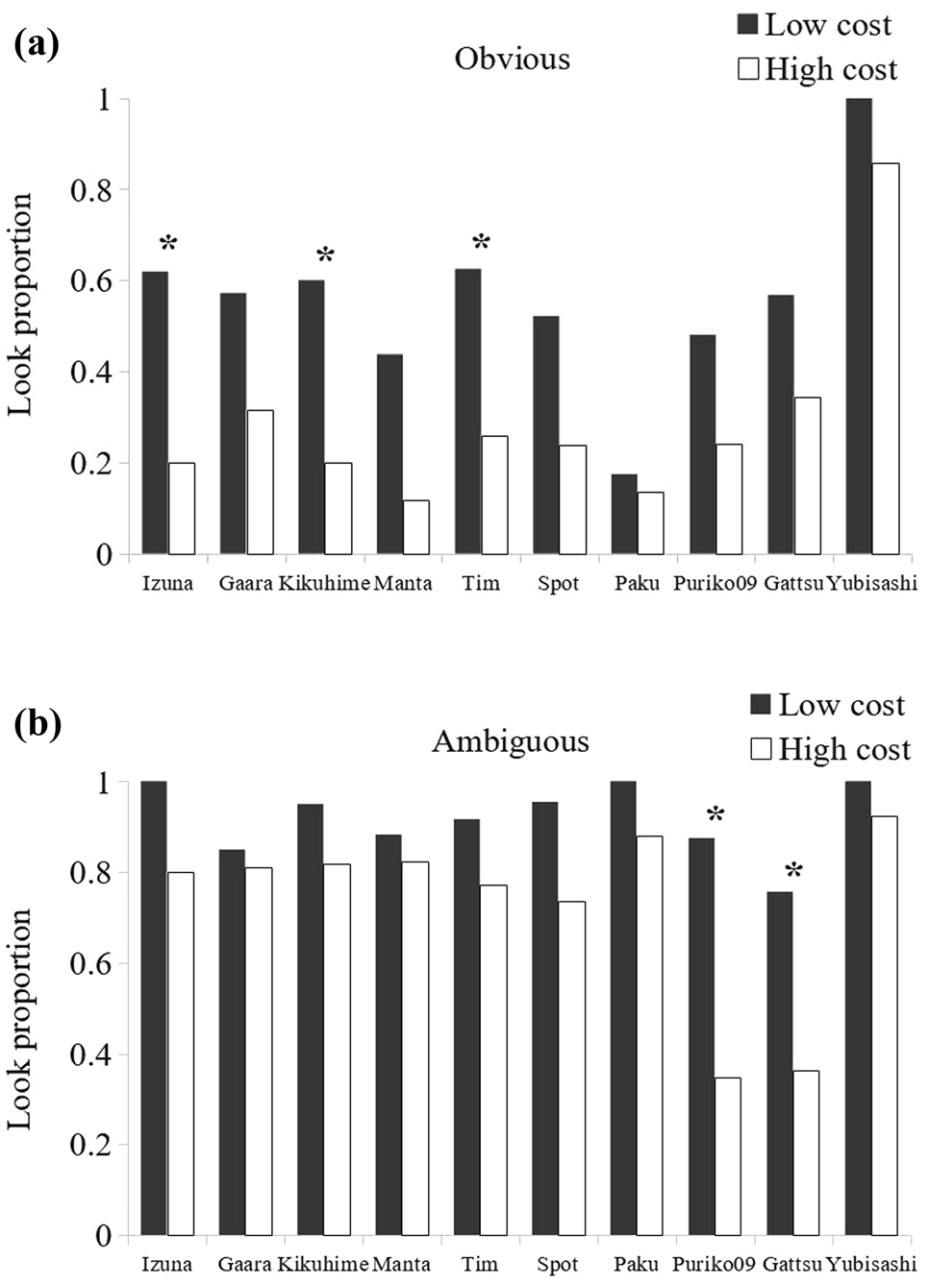
Proportion of looks made by each subject in low-cost condition (black) and high-cost condition (white), when the baiting was a. obvious or b. ambiguous. Stakes were always low. Data correspond to conditions 1 and 2 from Table 3

The effect of the cost of looking on the monkeys’ behavior was examined. At the group level, subjects were equally affected by the cost in both obvious and ambiguous conditions when stakes were low (i.e., only data from conditions 1 and 2 were compared). The overall mean proportion of looks decreased significantly as the cost to look increased (Fig. 5, obvious baiting: look at 56 ± 20% of trials for low cost, 29 ± 21% for high cost, Wilcoxon rank test: *Z* = − 2.75, *p* = 0.006; ambiguous baiting: look at 92 ± 08% of trials for low cost, 73 ± 20% for high cost, Wilcoxon rank test: *Z* = − 2.75, *p* = 0.006). At the individual level, five subjects showed a tendency to look inside the tubes less often when the cost of looking was high and baiting was obvious, but not when the baiting was ambiguous (Fig. 5). This difference was statistically significant for three of them (Table 4, Izuna: χ^2^(1.41) = 7.41, *p* = 0.006; Kikuhime: χ^2^(1.40) = 6.67, *p* = 0.01; Tim: χ^2^(1.51) = 6.93, *p* = 0.008; Gaara: χ^2^(1.40) = 2.63, *p* = 0.10; Manta: Fisher test, *p*(1.33) = 0.057). The differential effect of cost on the five participants contradicts the alternative hypothesis that they learned by association when looking and not looking (Table 5).

### Stakes effect

The effect of stakes on monkeys’ looking behavior was examined separately when the cost was low (Table 2, conditions 1 vs. 3) and high (Table 2, conditions 2 vs. 4). At the group level, we found no significant effect of stakes in either the obvious or ambiguous conditions, whether the cost was low or high (Table 3, Condition 1 vs. 3, Wilcoxon rank test: Obvious: *Z* = − 0.63, *p* = 0.53; Ambiguous: *Z* = − 0.059, *p* = 0.95; Condition 2 vs. 4, Wilcoxon rank test: Obvious: *Z* = − 1.33, *p* = 0.19; Ambiguous: *Z* = − 0.35, *p* = 0.73).

**Fig. 6:**
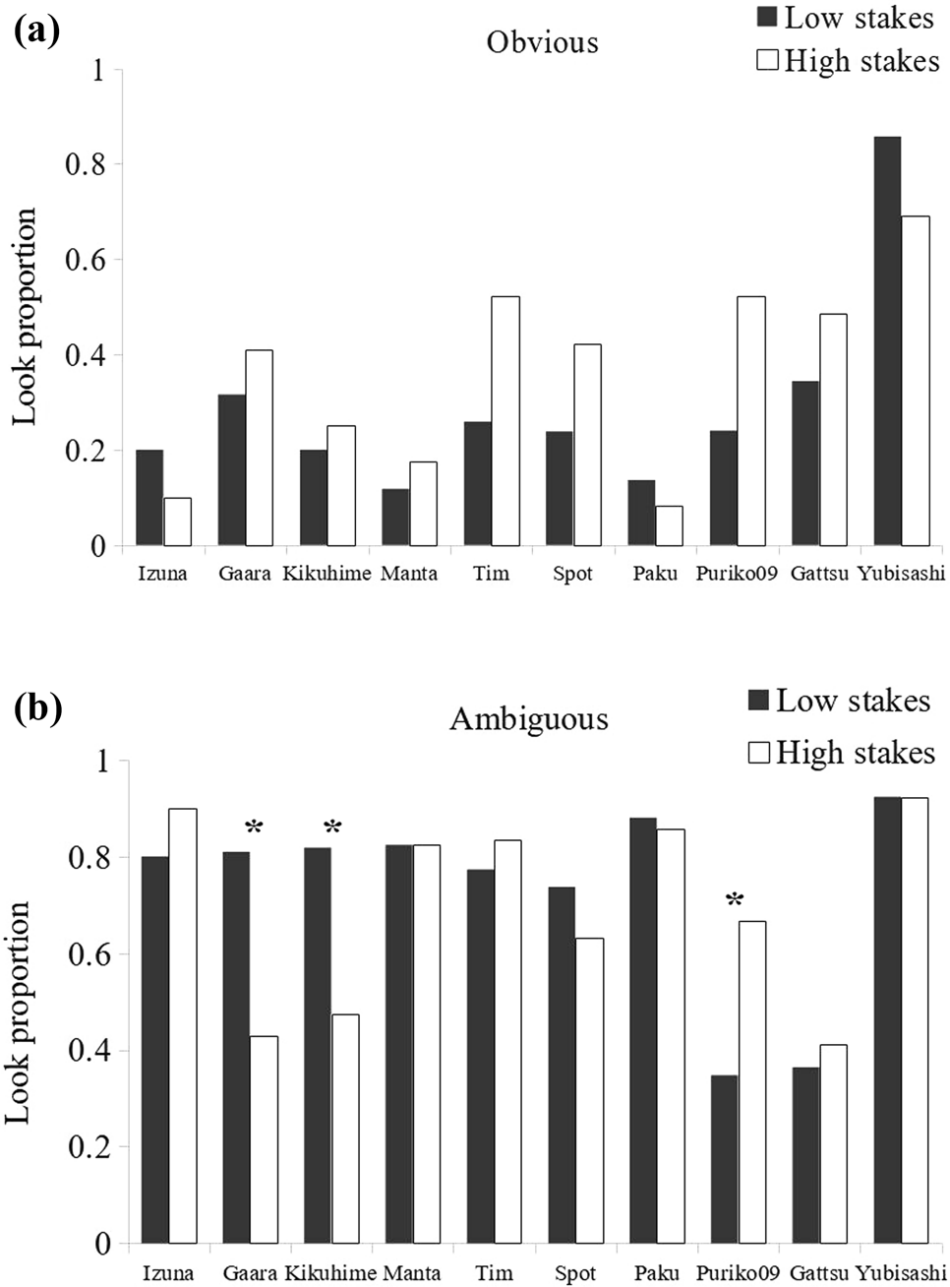
Proportion of looks made by each subject in low-stakes (black) and high-stakes (white) conditions, when the baiting was a. obvious or b. ambiguous. Only data from high-cost conditions (Table 3, data from conditions 2 and 4) are represented here

At the individual level, we observed some differences, although only a few were statistically significant (Table 4, Low vs. High Stakes). When the cost of looking was high, two subjects (Gaara and Kikuhime) looked significantly less often when the stakes were high, but they did so only in the ambiguous condition (Fig. 6; Gaara: *χ*^2^(1.42) = 6.46, *p* = 0.011; Kikuhime: *χ*^2^(1.41) = 5.38, *p* = 0.020). In the obvious condition, Gaara and Kikuhime showed no significant differences (Gaara: *χ*^2^(1.41) = 0.38, *p* = 0.54; Kikuhime: Fisher test, *p*(1.40) = 1.00). By contrast, four subjects (Tim, Spot, Puriko09 and Gattsu) showed a tendency to looked more inside the tubes when the stakes were high (Fig. 6, Table 3, condition 2 vs. 4). For three of them (Tim, Spot and Gattsu), this increase in looks was present only under the obvious condition. However, it does not reach statistical significance (Tim: *χ*^2^(1.50) = 3.63, *p* = 0.057; Spot: *χ*^2^(1.40) = 1.52, *p* = 0.22; Gattsu: *χ*^2^(1.69) = 1.44, *p* = 0.23). For Puriko09, the increase in looks appeared in both obvious and ambiguous baiting conditions, although the difference reached statistical significance only in the ambiguous condition (Obvious: *χ*^2^(1.48) = 1.46, *p* = 0.23; Ambiguous: *χ*^2^(1.47) = 4.78, *p* = 0.023).

When the cost to look was low, two subjects (Izuna and Gaara) showed the pattern predicted by the response competition hypothesis, looking 20 and 17% less often when the stakes were high and baiting was obvious (Table 3, condition 1 vs. 3). This decrease was not statistically significant (Low vs. high stake: Izuna: *χ*^2^(1.40) = 1.57, *p* = 0.21; Gaara:* χ*^2^(1.41) = 1.20, *p* = 0.27).

As it is possible that monkeys who look more often in the high-stakes condition do so because they may have more difficulty remembering the location of a high-value reward, we checked the influence of reward quality on monkeys’ success when baiting was obvious. We found no effect. Monkeys’ success was strictly identical (100% success) in low- and high-stakes conditions, regardless of them looking inside the tubes before selection. Only Gaara and Kikuhime made a mistake in the low-stakes condition without looking.

Our data provide little evidence to support the response-competition hypothesis. On the contrary, the tendency observed in four subjects (Tim, Spot, Puriko09 and Gattsu) who increased their looks when a high-quality reward was at stake directly contradicts the hypothesis, which anticipated a decrease in looking under the obvious condition (see Table 5 for a summary of alternative hypotheses predictions and results).

### Looks on obvious trials

**Fig. 7:**
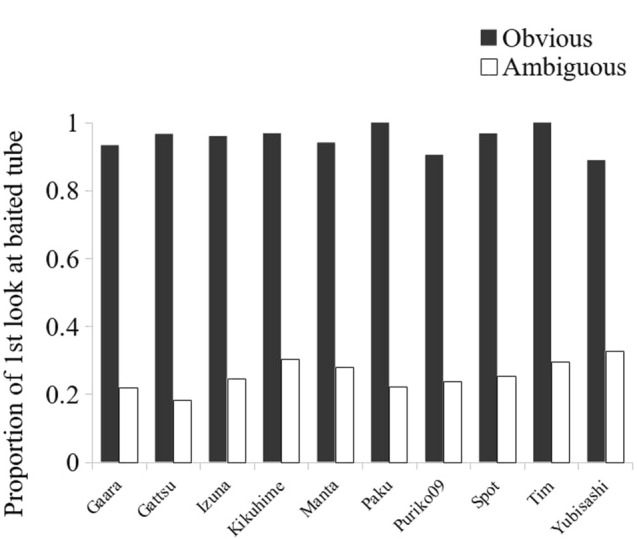
Proportion of trials in which each subject made his first look inside the baited tube when the baiting was obvious (black) and ambiguous (white)

Although the monkeys did not need to look inside the tubes to find the reward in the obvious condition, they looked at it on some occasions. Their first look was most often inside the baited tube (95 ± 04% of the trials ± SD, on average; Fig. 7). In the ambiguous condition, however, monkeys first looked inside the baited tube for only 26 ± 04% (SD) of the trials on average, which corresponds to the chance level. Therefore, when the monkeys looked in the obvious condition, they remembered the reward location. By contrast, they did not seem to know which tube contained the reward in the ambiguous condition.

### Search patterns

**Fig. 8:**
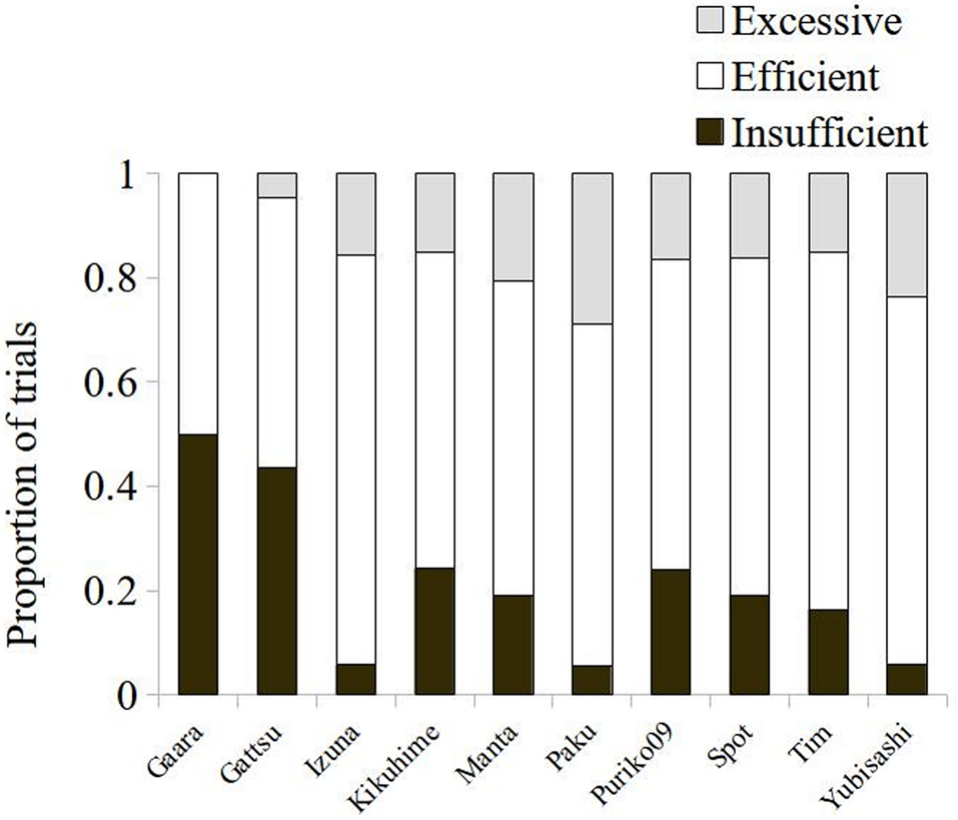
Proportion of trials in which each subject adopted an excessive (grey), efficient (white), or insufficient (black) search strategy when looking inside the tubes in the ambiguous condition. In the excessive strategy, monkeys had all the information needed to find the reward, but they kept looking (i.e., looked in the fourth tube after having looked inside three empty tubes or kept looking after having looked inside the baited tube). In the efficient strategy, monkeys ended their search as soon as all the information needed to make a correct choice was gathered (i.e., after spotting the reward inside a tube or having looked inside three empty tubes). In the insufficient strategy, monkeys ended their search before having enough information to make a correct choice (i.e., after looking inside one or two empty tubes only)

The search patterns used by the participants during the ambiguous trials were analyzed. Only trials in which the subjects looked at least once were included in the analysis. Friedman’s test revealed a significant difference between the three search patterns: efficient, insufficient, and excessive (*χ*^2^(2.10) = 14.21, *p* < 0.001). As a group, monkeys tended to adopt an efficient search strategy significantly more often (63 ± 09% of trials ± SD) than an insufficient (21 ± 15% of trials ± SD) or excessive one (16 ± 08% of trials ± SD) (efficient vs. insufficient: Wilcoxon rank test: *Z* = − 2.61, *p* = 0.009; efficient vs. excessive: Wilcoxon rank test: *Z* = − 3.10, *p* = 0.002). Importantly, most excessive looking can be attributed to a lack of inferential reasoning. 73 ± 32% of excessive looking trials were instances where monkeys could have inferred the location of the reward after having looked inside three empty tubes but checked the fourth tube. Instances where monkeys double checked an empty tube were very rare, only 13 ± 15% of excessive trials, and instances where monkeys kept looking after having looked in the baited tube were even rarer, 4 ± 5% of excessive trials. Thus, most subjects ended their search after spotting the reward. Only two (Gaara and Gattsu) adopted sufficient or insufficient search strategies in almost equivalent proportions (Fig. 8). In very few trials, monkeys did end their search after encountering three empty tubes, and selected the baited tube without checking its content, which could be a sign of inferential reasoning. This was observed in four subjects: Gattsu (4% of trials), Kikuhime (6%), Paku (1%), and Tim (2%).

Overall monkeys made appropriate use of the tubes by looking until they obtained the information needed to find the reward.

## Discussion

The goal of this experiment was to examine whether wild Japanese macaques would show the same tendencies as captive rhesus and lion-tailed macaques when tested in an information-seeking paradigm, that is, whether they would tend to seek more information when needed. If so, we aimed to clarify the cues and mechanisms that could underlie monkeys’ seeking behavior.

The primary finding of this study was that Japanese macaques discriminate between obvious and ambiguous trials; in most conditions, nine of our ten monkeys were significantly more likely to look inside the tubes before making a selection when baiting was ambiguous. Monkeys’ performances confirmed that they did not know which tube contained the food reward when baiting was ambiguous unless they looked inside the tubes. In addition, like apes and children (Call and Carpenter 2001; Gazes et al. 2023; Marsh and MacDonald 2012a, b), most of our monkeys were efficient in looking inside the tubes. That is, on average, they terminated their search as soon as they had the required information in 63% of the trials. However, unlike apes (Call and Carpenter 2001; Call 2010), macaques almost never used inference by exclusion when they had the chance. This difference between species could be explained by the number of tubes used: macaques were tested with four tubes, whereas apes were more often tested with two tubes. Macaques ability to infer the presence of food by exclusion was demonstrated with three possible locations (Marsh et al. 2015), but not four. It is also possible that information obtained through inference by exclusion is not accessible to metacognitive control in macaques. Our results are consistent with what has been previously observed in other species of macaques (Basile et al. 2015; Beran and Smith 2011; Brady and Hampton 2021; Hampton et al. 2004; Malassis et al. 2015; Marsh 2014) and suggests that Japanese macaques might be aware of their knowledge of hidden food.

Alternatively, monkeys may have learned by association when to look or not. Not only the hand movements were different between the obvious and ambiguous conditions, but also the time required to bait the tubes varied, which could have served as a cue for monkeys. Although the ambiguous baiting process was not introduced before the testing phase, and all subjects looked inside the tube during the first or second ambiguous trial (except for one individual, Paku), all subjects, except three (Izuna, Manta, and Yubisashi), took part in a preliminary study using two tubes and experienced a few ambiguous baiting trials. Thus, most of our monkeys had some opportunities to learn through association. Another possible explanation for monkeys metacognitive-like behavior would be response competition, as the metacognitive response (i.e., seeking-information by looking inside the tube) is in direct competition with the primary response (i.e., selecting a tube).

In an attempt to clarify this, we manipulated the cost of looking and the stakes of the experiment as was done with apes (Call 2010; Gazes et al. 2023; Marsh and MacDonald 2012b; Mulcahy 2016), and found that half of the subjects reacted by decreasing unnecessary looks whenever we increased the cost of looking, whereas four tended to increase unnecessary looks when a preferred food item was at stake. These results challenge the associative learning and response–competition hypotheses (Table 5).

### Cost and stakes effects

We hypothesized that if monkeys’ looking behavior is guided by associative learning mechanisms, they should look at the same frequency regardless of the cost and stakes, or if cost and stakes have an effect on look proportions, it should have an equal effect in obvious and ambiguous conditions. However, if monkeys’ looking behavior was guided by response competition, we expected the monkeys to look less when the stakes were higher in the obvious condition, where the location of the reward was known (Table 5). If we found strong consistency in their behavior when comparing obvious and ambiguous conditions, manipulating the cost and stakes reveals great inter-individual variability.

Among the nine monkeys who showed metacognitive-like behavior, a differential effect of cost was found in five; that is, they tended to look less when the cost was high in the obvious condition. This difference was statistically significant for three of them (Izuna, Kikuhime, and Tim). However, the absence of statistical significance in the other two cases (Gaara and Manta) does not necessarily indicate that the observed difference was random, as the number of trials may have been insufficient to detect a meaningful effect. By contrast, these five monkeys kept looking at a high frequency when looking was necessary in the ambiguous condition. Similarly, three (Tim, Spot and Gattsu) were affected by stakes in a way that challenged the associative learning account, looking more when a high-quality reward was at stake in the obvious baiting condition. Although the difference did not reach statistical significance, we would argue this is a meaningful difference because, in Tim and Spot cases, the frequency of their looking doubled. As for Gattsu, it is important to note that he did not look significantly more often in the ambiguous compared to obvious baiting condition with a low-quality reward, but did when a high-quality reward was at stake, granted that the cost was low. This denotes an effect of stakes on his looking behavior.

Thus, seven of our nine monkeys who displayed metacognitive-like behavior showed differential effects of cost, stakes, or both, which seems to indicate that they are not simply using external cues, such as hand movements or baiting time, to decide when to look. It is more difficult to evaluate the extent to which the two other monkeys relied on stimulus configurations. However, by controlling for associative learning, the last decade of studies has brought about a strong consensus that the performance seen in many tests of animal metacognition is unlikely to be conditioned by external cues and the results of associative learning only (Brady and Hampton 2021; Beran 2019; Fujita 2009; Hampton 2019). Taken together with previous studies on monkeys, we believe that associative learning is not a satisfactory explanation for most of our monkeys’ behaviors.

The response competition hypothesis is often considered a plausible explanation and some researchers have argued that it does not necessarily deny memory awareness (Hampton et al. 2004). In this study, the pattern predicted by the response competition hypothesis was observed in Izuna and Gaara; however, given the small difference and lack of statistical significance, it is difficult to determine whether this observation is meaningful. The only undeniable decrease in the look proportion was that observed in Gaara and Kikuhime in the ambiguous baiting condition. When the cost of looking was low, Gaara and Kikuhime continued looking at a high frequency in ambiguous baiting trials, even when using a high-quality reward. Therefore, the decrease they show in the high-cost/high-stakes condition seems to be a combined effect of increased costs and stakes. This result does not align with the response-competition hypothesis, which predicted an increased difference in look proportions between obvious and ambiguous conditions. Instead, it appears that an excessively appealing reward, coupled with a high cost, may have led some monkeys to refrain from looking, regardless of whether they were presented with an obvious or ambiguous baiting condition.

Overall, our data provide little evidence to support the response-competition hypothesis. By contrast, our observations tend to go in the opposite direction, with the use of a high-quality reward leading to an increase in look frequency for the four subjects. The others did not appear to be affected by the quality of the reward under any condition. It could be that the rewards we used for the low- and high-stakes conditions were of equal value to them, or that they might have been very motivated to obtain food, regardless of the type.

Finally, there were three subjects (Gattsu, Tim and Spot) whose tendencies contradicted both the associative learning and response competition hypotheses (Table 5). Non-metacognitive accounts based on postulating curiosity as a mediating variable seem more successful in explaining the current data.

### Curiosity and inhibition

Recently, Carruthers and Williams (2019) argued that the differential looking frequencies observed in apes and monkeys tested in information-seeking experiments could be best explained by first-order questioning attitudes (Carruthers 2008; Friedman 2013; Whitcomb 2010). They defined curiosity as an affective emotion-like motivating state that takes the form of a question, such as, *where is the food?* This questioning attitude will drive the animal to act to satisfy their feeling of curiosity, for example, by engaging in a search (the same way fear will lead to the impetus to run). Therefore, a monkey that has seen food hidden in one of several potential locations without seeing it will be prompted into a questioning state with the content, *where is the food?* According to Carruthers and Williams, monkeys are sensitive to salient forms of ignorance but do not represent ignorance. In this view, it is also conceivable that animals might feel curious until they have gathered the right amount of information, or the relevant information, to reach a decision, which could explained results like those of Beran et al. (2013) or Brandy and Hampton (2021).

However, in the present study, if monkeys looked significantly more often in the ambiguous condition than in the obvious condition, they looked in the obvious condition as well, in 44% of the trials on average, and their first look was almost always directed toward the baited tube. Therefore, it seems that the monkeys remembered the location of the reward but looked anyway. Call (2010) proposed that apes look in the “known” condition because they might want to secure the reward and be sure that they remember well (passport effect). As Call and Carpenter (2001) proposed, if the cost of looking is too small, the benefit to be gained from doing so outweighs the cost incurred, even if it is just to check the reward’s location.

Alternatively, subjects may have been guided by a feeling of curiosity (or uncertainty) and looked inside the tubes during obvious trials due to difficulty in inhibiting impulsive looking behavior. It has been suggested that looking at a reward may be attractive in itself (Perner 2012). The use of a more appealing reward would make it even more difficult, whereas an increased cost would make it easier for monkeys to refrain from looking. To advance this debate, this question needs to be addressed in future studies.

### Ambiguous baiting procedure

Our study is unique in that it employs an ambiguous baiting procedure rather than an opaque panel to create “unknown” trials. Our results demonstrate that the ambiguous baiting procedure we utilized is a valid method for generating “unknown” trials. However, it is crucial to acknowledge its limitations; the manipulation demands a certain level of dexterity and results in a time discrepancy depending on the baiting procedure, which could serve as a cue for associative learning. Additional subtle cues could also assist the monkeys in detecting the reward location. Ambiguous baiting does not necessarily create a situation where the subject has absolutely no idea where the reward is; instead, it induces a state of uncertainty, the intensity of which can vary. Depending on the study's objectives, this uncertainty could be an intriguing aspect to explore. It might be worthwhile to investigate whether monkeys exhibit reduced looking when presented with an ambiguous baiting process in comparison to an opaque panel.

### To conclude

This study supports the idea that monkeys are able to adaptively monitor their behavior based on their knowledge state, and that this ability is shared in the Macaca genus. Additionally, it shows that the tube task can be implemented in the wild while controlling for a variety of factors, providing useful data for comparisons between laboratory-housed and free-ranging animals. Confirming the observations of Rosati and Santos (2016), our results revealed similar abilities between free-ranging and captive macaques, demonstrating that the metacognitive-like performances observed in the laboratory are not the result of extensive training. Taken together with previous work, our study emphasizes that the mere presence or absence of memory is not the only factor controlling monkeys’ decisions to seek information. Monkeys sometimes seek information on the location of food even though they already possess a memory. Moreover, some subjects appeared to be sensitive to the cost of seeking and/or value of the reward. Similar to the findings in apes (Call 2010), some macaques reduced unnecessary looking when the cost of looking was high, while others increased their looking behavior when a high-quality food item was at stake. However, only one macaque was affected by both cost and stakes in this way, and unlike in apes, this behavioral pattern was not evident when analyzing macaque group behavior. This disparity suggests that the behavioral flexibility observed in great apes might not be as prevalent among macaques. Alternatively the variability observed between our macaques could be a matter of personality (some subjects were probably more willing to take risks or make efforts). Or the parameters we chose to manipulate (i.e., type of food and apparatus height) might have failed to reveal an effect in some monkeys. Finally, this could indicate that the cognitive mechanisms underlying our macaques’ seeking behavior were not the same for each subject. Is it possible that only some subjects of a species are able to (or willing to) use metacognition, especially if its benefit is not very high? Smith (2005) noted that both humans and other primates tested on escape response paradigm display similar ranges of individual differences, with some people and animals who never used the “escape” option.

Nevertheless, monkeys appropriately refrained from looking when looking can be dispensed, looked when looking is required, and were effective in the way they did. Whether these results reflect “true” memory awareness remains debatable. Several non-metacognitive hypotheses have been proposed to explain animals’ looking responses, but the current data do not neatly fit the predictions of these hypotheses. One observation that could make a strong argument in favor of metacognition is that monkeys still look from time to time, even though they know the reward’s location, and some tend to do it more when the stakes are high. Kornell (2013) suggests that metacognitive errors provide strong evidence of animal and human metacognition. However, whether the looks observed in the obvious/known condition reflect metacognitive errors, a desire to confirm one’s knowledge (i.e., the “passport effect”), or an inability to inhibit foraging behavior remains unclear. Testing individuals’ ability to inhibit looking/reaching for food responses and checking for a negative correlation between monkeys’ look frequency in the obvious condition and their inhibition capabilities, may shed some light on this. The relationship between metacognitive monitoring and inhibitory control abilities requires further investigation.

## Supplementary Information

Below is the link to the electronic supplementary material.

**Supplementary file 1. Online Resource 1**. Food preferences. Monkeys food preferences were assessed during the course of the training phase. To do so, the different types of food (carrot, sweet potato and peanut) were presented in pairs to the subject who had to choose one. Because monkeys preferences could varied from day to day, food preference tests were carried out over several days during the training period and monkeys received no more than five trials a day, until each pair was presented a minimum of 12 times. The most frequently selected item was retained for use as a high-quality reward, while the least frequently selected item was used as a low-quality reward (provided it was sufficiently appreciated to motivate the monkey to participate in the experiment; otherwise, the moderately appreciated item was used). Once established, the subjects' preferences were checked by two or three trial runs before the tests began. (DOCX 14 KB)

**Supplementary file 2** (DOCX 15 KB)

## Data Availability

All data analyzed in this study are included in the published article and its supplementary information files.
