# Supplementary material for: Metacognition in wild Japanese macaques: cost and stakes influencing information-seeking behavior
Source: Anim Cogn. 2024 Mar 5;27(1):22. doi: 10.1007/s10071-024-01851-z (PMC10914859; doi:10.1007/s10071-024-01851-z)
Supplement: Supplementary file 1 — Online Resource 1. Food preferences. Monkeys food preferences were assessed during the course of the training phase. To do so, the different types of food (carrot, sweet potato and peanut) were presented in pairs to the subject who had to choose one. Because monkeys preferences could varied from day to day, food preference tests were carried out over several days during the training period and monkeys received no more than five trials a day, until each pair was presented a minimum of 12 times. The most frequently selected item was retained for use as a high-quality reward, while the least frequently selected item was used as a low-quality reward (provided it was sufficiently appreciated to motivate the monkey to participate in the experiment; otherwise, the moderately appreciated item was used). Once established, the subjects' preferences were checked by two or three trial runs before the tests began. (DOCX 14 KB) [file 10071_2024_1851_MOESM1_ESM.docx]

**Metacognition in wild Japanese macaques: cost and stakes influencing information-seeking behavior.**

*Animal Cognition*

Lorraine Subias^1^, Noriko Katsu^1^, Kazunori Yamada^1^

^1^Graduate School of Human Sciences, Osaka University

1-2 Yamadaoka, Suita, Osaka, Japan

+ 81 80 9530 4081

subias.lorraine@hotmail.fr

**Online Resource 1**

**Food preferences**

Monkeys food preferences were assessed during the course of the training phase. To do so, the different types of food (carrot, sweet potato and peanut) were presented in pairs to the subject who had to choose one. Because monkeys preferences could varied from day to day, food preference tests were carried out over several days during the training period and monkeys received no more than five trials a day, until each pair was presented a minimum of 12 times. The most frequently selected item was retained for use as a high-quality reward, while the least frequently selected item was used as a low-quality reward (provided it was sufficiently appreciated to motivate the monkey to participate in the experiment; otherwise, the moderately appreciated item was used). Once established, the subjects' preferences were checked by two or three trial runs before the tests began.
