# Supplementary material for: Metacognition in wild Japanese macaques: cost and stakes influencing information-seeking behavior
Source: Anim Cogn. 2024 Mar 5;27(1):22. doi: 10.1007/s10071-024-01851-z (PMC10914859; doi:10.1007/s10071-024-01851-z)
Supplement: Supplementary file 2 — (DOCX 15 KB) [file 10071_2024_1851_MOESM2_ESM.docx]

**Metacognition in wild Japanese macaques: cost and stakes influencing information-seeking behavior.**

*Animal Cognition*

Lorraine Subias^1^, Noriko Katsu^1^, Kazunori Yamada^1^

^1^Graduate School of Human Sciences, Osaka University

1-2 Yamadaoka, Suita, Osaka, Japan

+ 81 80 9530 4081

subias.lorraine@hotmail.fr

**Online Resource 2**

| **Binomial tests results** | | | |
| --- | --- | --- | --- |
|  | **Nb of success** | **Nb of trials** | **p-value** |
| **Gaara** | 7 | 32 | 0.84 |
| **Gattsu** | 30 | 87 | 0.047 |
| **Izuna** | 7 | 25 | 0.82 |
| **Kikuhime** | 8 | 39 | 0.58 |
| **Manta** | 4 | 22 | 0.62 |
| **Paku** | 2 | 23 | 0.090 |
| **Puriko09** | 12 | 48 | 1 |
| **Spot** | 8 | 36 | 0.85 |
| **Tim** | 11 | 33 | 0.31 |
| **Yubisashi** | 8 | 19 | 0.12 |

Results from binomial tests to assess if monkeys performed above chance level in the ambiguous condition when they did not look inside the tubes. Probability of success was 0.25.
